# Predictive value of high-sensitivity troponin-I for future adverse cardiovascular outcome in stable patients with type 2 diabetes mellitus

**DOI:** 10.1186/1475-2840-13-63

**Published:** 2014-03-25

**Authors:** Kai-Hang Yiu, Kui-Kai Lau, Chun-Ting Zhao, Yap-Hang Chan, Yan Chen, Zhe Zhen, Arthur Wong, Chu-Pak Lau, Hung-Fat Tse

**Affiliations:** 1 Cardiology Division, Department of Medicine, Queen Mary Hospital, the University of Hong Kong, Block K, Pokfulam, Hong Kong; 2 Research Centre of Heart, Brain, Hormone and Healthy Aging, Li Ka Shing Faculty of Medicine, the University of Hong Kong, Hong Kong, SAR, China; 3 Division of Neurology, Department of Medicine, Queen Mary Hospital, the University of Hong Kong, Hong Kong, SAR, China

**Keywords:** Type 2 diabetes mellitus, High-sensitivity troponin I outcome

## Abstract

**Introduction:**

High-sensitivity cardiac troponin I(hs-TnI) and T levels(hs-TnT) are sensitive biomarkers of cardiomyocyte turnover or necrosis. Prior studies of the predictive role of hs-TnT in type 2 diabetes mellitus(T2DM) patients have yielded conflicting results. This study aimed to determine whether hs-TnI, which is detectable in a higher proportion of normal subjects than hsTnT, is associated with a major adverse cardiovascular event(MACE) in T2DM patients.

**Methods and results:**

We compared hs-TnI level in stored serum samples from 276 consecutive patients (mean age 65 ± 10 years; 57% male) with T2DM with that of 115 age-and sex-matched controls. All T2DM patients were prospectively followed up for at least 4 years for incidence of MACE including heart failure(HF), myocardial infarction(MI) and cardiovascular mortality. At baseline, 274(99%) patients with T2DM had detectable hs-TnI, and 57(21%) had elevated hs-TnI (male: 8.5 ng/L, female: 7.6 ng/L, above the 99^th^ percentile in healthy controls). A total of 43 MACE occurred: HF(n = 18), MI(n = 11) and cardiovascular mortality(n = 14). Kaplan-Meier analysis showed that an elevated hs-TnI was associated with MACE, HF, MI and cardiovascular mortality. Although multivariate analysis revealed that an elevated hs-TnI independently predicted MACE, it had limited sensitivity(62.7%) and positive predictive value(38.5%). Contrary to this, a normal hs-TnI level had an excellent negative predictive value(92.2%) for future MACE in patients with T2DM.

**Conclusion:**

The present study demonstrates that elevated hs-TnI in patients with T2DM is associated with increased MACE, HF, MI and cardiovascular mortality. Importantly, a normal hs-TnI level has an excellent negative predictive value for future adverse cardiovascular events during long-term follow-up.

## Introduction

Cardiac troponin is a key biomarker for the diagnosis of myocardial infarction (MI) and provides important prognostic information in patients who present with acute coronary syndrome [1-3]. High-sensitivity cardiac troponin levels T (hs-TnT) and I (hs-TnI) are more sensitive biomarkers that can detect troponin below the clinical threshold, and reflect subtle cardiomyocyte turnover or necrosis. These novel biomarkers have been shown to be associated with adverse clinical and cardiovascular events in the general population [4-7], and in patients with congestive heart failure [8], acute coronary syndrome [9] and stable atherosclerotic disease [10,11].

Recent studies have demonstrated that patients with type 2 diabetes mellitus (T2DM) without prior cardiovascular disease [12] and a high hbA1c% had elevated hs-TnT [13]. It has been proposed that chronic hyperglycemia contributes to subtle myocardial injury as detected by hs-TnT that is beyond its effects on development of clinical atherosclerotic coronary disease [13]. The clinical implications of an elevated high-sensitivity troponin assay in patients with T2DM nonetheless remain unclear. Hallen et al. [14] showed that elevated hs-TnT was frequently detected in diabetic patients but did not predict future adverse outcomes over a 2 year follow-up period. Conversely, recent results from the Women’s Health Study demonstrated that a detectable level of hs-TnT was associated with increased cardiovascular morbidity and mortality in diabetic women [12]. Compared with hs-TnT measurement, higher proportions of normal subjects have a detectable level of hs-TnI, making it a more sensitive assay for subtle myocardial damage [15]. Currently, there are no data on the predictive value of hs-TnI in patients with T2DM. This study sought to investigate whether an elevated hs-TnI is associated with a major adverse cardiovascular event (MACE) in patients with T2DM.

## Methods

### Study population

Consecutive T2DM patients (n = 293) as defined by World Health Organization criteria [16,17] on stable hypoglycemic and cardiovascular medication for at least 3 months were recruited from the medical outpatient clinic. Exclusion criteria included recent acute coronary syndrome, stroke, coronary intervention, hospitalization for cardiac surgery or heart failure within the last 6 months, dilated cardiomyopathy, significant valvular heart disease, chronic atrial fibrillation, New York Heart Association class III/IV heart failure, estimated glomerular filtration rate (eGFR) <30 mL/min per 1.73 m2, and refusal to participate (n = 17). A total of 276 patients with T2DM were consequently eligible for this study. During the study period, 115 age- and sex-matched Chinese controls without T2DM or established cardiovascular disease were recruited from a community health screening programme. Written informed consent was obtained from all study subjects. The study was approved by the Institutional Review Board of the University of Hong Kong/Hospital Authority Hong Kong West Cluster and was conducted according to the Declaration of Helsinki. This study is part of the Chinese Diabetic Heart Study (CDATS) to evaluate cardiovascular manifestation of Chinese patients with T2DM, in an attempt to evaluate the pathophysiology and potential therapeutics in these patients.

### Clinical parameters

Baseline demographic data, clinical characteristics and blood sampling were obtained on the same day from all study subjects. Blood pressure, body weight, body height, and body mass index (BMI) were also measured. Hypertension was defined as resting systolic or diastolic blood pressure >140 mmHg or >90 mm Hg, respectively, at two different clinic visits or the prescription of antihypertensive medication. Hypercholesterolemia was defined as fasting total plasma cholesterol ≥4.9 mmol/liter or the prescription of statins. Smoking status was recorded as ever smoker (past or current) or nonsmoker. Duration of T2DM and data on prescribed oral hypoglycemic agents and insulin therapy were retrieved from patients’ medical records.

Serum HbA1c, total cholesterol, triglyceride, high-density lipoprotein cholesterol and low-density lipoprotein cholesterol levels, fasting glucose, and HbA1c were measured in all subjects in a fasting venous blood sample [18]. Serum creatinine levels were used to assess eGRF calculated with the Modified Diet in Renal Disease Equation [19]. Serum level of hs-TnI was determined using Chemoluminescent Microparticule ImmunoAssay (Architect i1000SR Abbott®, Paris, France). The level of detection is 1.2 ng/L according to the manufacturer’s instruction and above such is considered to be a detectable hs-TnI. An elevated hs-TnI was defined as plasma level greater than the 99^th^ percentile based on the hs-TnI of an age-matched healthy control for both genders, respectively.

### Follow-up

All patients were followed up for a minimum of 4 years. Outcome of patients was retrieved from the inter-hospital computer system or by telephone interview. The MACE was a composite endpoint of heart failure requiring hospital admission, myocardial infarction and cardiovascular mortality. The definition of MI was based on the presence of typical chest pain, elevated cardiac enzyme levels, and typical electrocardiogram changes [1].

### Statistical analysis

Data are expressed as mean ± standard deviation for continuous variables and frequencies or proportions for categorical variables. Continuous demographic variables of the two groups were compared using the Mann-Whitney *U* test and categorical demographic variables compared using Pearson Chi-square test or the Fisher’s exact test if at least one cell had an expected cell count below five. Cumulative incidence of the first occurrence of MACE for patients with elevated hs-TnI and normal hs-TnI level was estimated using the Kaplan-Meier method and compared with the log-rank test. First occurrence of heart failure, myocardial infarction and cardiovascular mortality was evaluated. Multivariate analyses for MACE, heart failure, myocardial infarction and cardiovascular mortality were performed using Cox regression models.

Three levels of adjustment were made: (1) demographics (age and gender); (2) demographic factors, cardiovascular risk factors (hypertension, hyperlipidemia, smoking history, coronary heart disease); (3) demographic factors, risk factors, cardiovascular risk factors and eGFR level. All statistical analyses were performed using the statistical package SPSS for windows (Version 18.0, SPSS, Chicago, USA). All P values reported are 2-sided for consistency. A *P value <0.05* was considered statistically significant.

## Results

### Clinical characteristics

Baseline characteristics of patients with T2DM and controls are shown in Table 1. Patients with T2DM had a higher BMI, were more likely to be a smoker and had a history of hypertension and hypercholesterolemia compared with controls. In addition, the eGFR was lower, and fasting glucose and HbA1c% were higher.

**Table 1:** Baseline demographics of type 2 diabetes mellitus (T2DM) patients with and without elevated high sensitivity Troponin I (hs-TnI) and controls

**Parameters**	**T2DM (n = 276)**	**Controls (n = 115)**	**P value**	**Elevated hs-TnI (n = 70)**	**Normal hs-TnI (n = 206)**	**P value**
Age, years	64.4 ± 10.0	63.4 ± 7.9	0.29	68.6 ± 9.2	62.9 ± 9.9	<0.01
Male, % (n)	154 (56)	53 (61)	0.35	70 (49)	30 (21)	<0.01
Body mass index, kg/m^2^	25.5 ± 3.5	23.6 ± 3.4	<0.01	25.4 ± 3.5	25.6 ± 3.6	0.72
Current smoker, % (n)	33 (92)	6 (7)	<0.01	49 (34)	28 (58)	<0.01
Hypertension, % (n)	70 (194)	15 (17)	<0.01	89 (62)	132 (64)	<0.01
Hypercholesterolemia, % (n)	63 (174)	30 (26)	<0.01	70 (49)	61 (125)	0.10
Duration of DM, years	9.8 ± 7.6	--	--	10.2 ± 8.7	9.7 ± 7.3	0.67
CAD, % (n)	29 (113)	--	--	60 (42)	35 (71)	<0.01
Total cholesterol, mmol/L	4.6 ± 1.0	5.0 ± 0.9	<0.01	4.5 ± 1.1	4.7 ± 1.0	0.15
Triglycerides, mmol/L	1.6 ± 1.8	1.3 ± 0.8	<0.01	1.6 ± 1.1	1.6 ± 1.9	0.99
High density lipoprotein, mmol/L	1.3 ± 0.8	1.5 ± 0.4	<0.01	1.2 ± 0.4	1.3 ± 0.4	0.16
Low density lipoprotein, mmol/L	2.7 ± 0.8	3.0 ± 0.7	<0.01	2.6 ± 0.8	2.7 ± 0.8	0.55
eGFR, mL/min per 1.73 m2	81.8 ± 19.3	85.8 ± 14.1	0.03	72.0 ± 18.7	84.2 ± 18.7	<0.01
Fasting glucose, mmol/L	7.6 ± 2.3	5.1 ± 0.5	<0.01	7.6 ± 2.8	7.6 ± 2.1	0.96
HbA1c, %	7.8 ± 1.4	5.9 ± 0.4	<0.01	8.1 ± 1.7	7.7 ± 1.4	0.21
**Medication**						
Insulin, % (n)	14 (38)	0 (0)	<0.01	19 (13)	12 (25)	0.13
Aspirin, % (n)	40 (109)	0 (0)	<0.01	56 (39)	34 (70)	<0.01
ACEI/ARB, % (n)	60 (166)	1 (1)	<0.01	77 (54)	54 (112)	<0.01
Statin, % (n)	42 (115)	2 (2)	<0.01	57 (40)	37 (75)	<0.01

*Abbreviation*:

ACEI = angiotensin converting enzyme inhibitor; ARB = angiotensin receptor blocker; CAD = coronary artery disease; eGFR = estimated glomerular filtration rate.

### Serum level of hs-TnI

The proportion of patients with T2DM and serum level of hs-TnI at or above the limit of detection (1.2 ng/L) was similar to controls (274/276, 99.3% versus 114/115, 99.1%, *P = 1.0*). The median serum level of hs-TnI in patients with T2DM was significantly higher (median [interquatile range]: 4.8 [3.2-8.4 ng/L] versus 2.9 [2.2-3.9 ng/L], *P < 0.01*).

In this study, the 99^th^ percentile value of serum hs-TnI level in male and female control subjects was 8.5 ng/L and 7.6 ng/L, respectively. These serum levels were defined as the cut-off values for elevated serum hs-TnI. Based on these cut-off values, 70 (25.4%) patients with T2DM had an elevated serum hs-TnI level. As shown in Table 1, T2DM patients with elevated serum hs-TnI level were older, more likely to be male, smoke, have a history of hypertension and coronary artery disease, low eGFR level, and be treated with aspirin, angiotensin converting enzyme inhibitor/angiotensin receptor blocker and statin compared with T2DM patients with a normal serum hs-TnI level. Univariate analysis showed that elderly age, male gender, smoking, a history of hypertension and coronary artery disease and low eGFR were associated with elevated serum hs-TnI level in T2DM patients. Multivariate analysis nonetheless revealed that only history of coronary artery disease and low eGRF were independently associated with an elevated serum hs-TnI level (Table 2).

**Table 2:** Predictors for high-sensitivity troponin I in patients with type 2 diabetes mellitus

	**Univariate analysis**			**Multivariate analysis**		
**Variables**	**β**	**95% CI**	**P value**	**β**	**95% CI**	**P value**
Age	1.07	1.03-1.10	<0.01	1.02	0.97-1.06	0.46
Male gender	2.24	1.26-4.01	<0.01	2.08	0.82-5.28	0.12
Body mass index	0.99	0.91-1.07	0.72			
Smoker	2.41	1.38-4.21	<0.01	1.58	0.64-3.87	0.32
Hypertension	4.35	1.97-9.57	<0.01	2.21	0.90-5.44	0.09
Duration of disease	1.01	0.97-1.05	0.64			
History of CAD	2.85	1.63-4.98	<0.01	2.93	1.33-6.44	<0.01
Total Cholesterol	0.80	0.60-1.08	0.14			
Triglyceride	0.99	0.84-1.19	0.99			
High density lipoprotein	0.51	0.20-1.27	0.15			
Low density lipoprotein	0.89	0.60-1.31	0.54			
eGFR	0.97	0.95-0.98	<0.01	0.97	0.95-0.99	0.02
Fasting glucose	0.99	0.88-1.13	0.96			
HbA1c	1.17	0.94-1.45	0.16			
Insulin	1.65	0.79-3.44	0.18			

Abbreviations as in Table 1.

### Clinical outcomes

**Figure 1:**
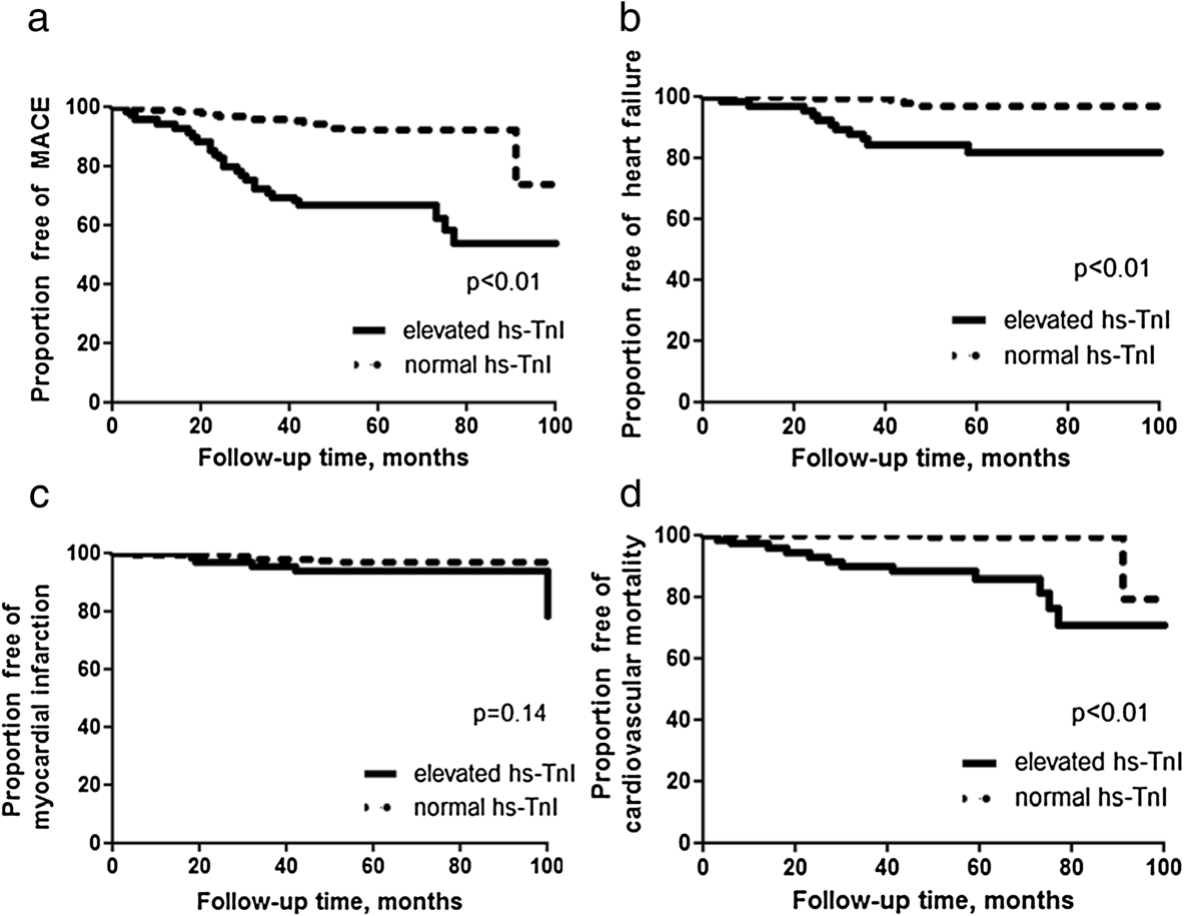
Kaplan-Meier Curve reflecting cumulative proportion of patients with type 2 diabetes mellitus free of (a) major adverse cardiovascular events [MACE]; (b) heart failure; (c) myocardial infarction; and (d) cardiovascular mortality.

The median follow-up period was 4.9 years (interquartile range, 3.7 to 5.6 years), and none of the control subjects developed MACE. A total of 43 patients with T2DM developed MACE during this follow-up period. Among those MACEs, there were 18 heart failure events (12 diastolic heart failure, 6 systolic heart failure), 11 myocardial infarctions and 14 cases of cardiovascular mortality (11 systolic heart failure, 2 myocardial infarction and 1 sudden death). For the whole population, the annual MACE event rate was 3.3%, heart failure event rate was 1.4%, myocardial infarction event rate was 1.3% and cardiovascular mortality event rate was 1.0%. More importantly, T2DM patients with elevated hs-TnI had a higher annual event rate for MACE (9.3% vs. 1.6%), heart failure (3.8% vs.0.6%), myocardial infarction (1.5% vs.0.6%) and cardiovascular mortality (3.5% vs. 0.2%) than those T2DM patients with normal serum hs-TnI level (*P < 0.01*). As shown in Figure 1, T2DM patients with elevated serum level of hs-TnI had a significantly higher risk for MACEs (Hazard ratio [HR] 8.9, 95% confidence interval [CI] 4.3-18.4 , P < 0.01); heart failure (HR 19.6, 95% CI 4.0-40.6 , P < 0.01), and cardiovascular mortality (HR 17.1 95% CI 5.3-55.5 , P < 0.01), but not myocardial infarction (HR 2.9, 95% CI 0.7-11.5, P = 0.14) than those with normal serum hs-TnI level during follow-up.

### Predictive value of serum hs-TnI level

Cox proportional hazard models were used to evaluate the impact of elevated serum hs-TnI level on adverse clinical outcome (Table 3). In both the unadjusted model and adjusted models for demographic factors and cardiovascular risk factors, an elevated serum hs-TnI level in patients with T2DM was predictive of MACE, heart failure and cardiovascular mortality. Even after adjusting for demographic factors, cardiovascular risk factors and eGFR, an elevated serum hs-TnI level remained an independent predictor for MACE and heart failure.

**Table 3:** Association of elevated high-sensitivity troponin I (hs-TnI) with subsequent major adverse cardiovascular events (MACE), heart failure, myocardial infarction and cardiovascular mortality

	**Univariate analysis**		
**Variables**	**HR**	**95% CI**	**P value**
MACE			
Unadjusted^a^	5.69	3.05-10.62	<0.01
Adjusted for demographic factors^b^	3.81	2.01-7.24	<0.01
Adjusted for demographic, cardiovascular risk factors^c^	3.70	1.91-7.18	<0.01
Adjusted for demographic, cardiovascular risk factors and eGFR^d^	2.85	1.15-7.03	0.02
Heart failure			
Unadjusted^a^	7.21	2.70-19.25	<0.01
Adjusted for demographic factors^b^	4.98	1.80-13.83	<0.01
Adjusted for demographic, cardiovascular risk factors^c^	4.88	1.71-13.96	<0.01
Adjusted for demographic, cardiovascular risk factors and eGFR^d^	4.88	1.12-21.31	0.03
Myocardial infarction			
Unadjusted^a^	2.45	0.72-8.31	0.15
Adjusted for demographic factors^b^	1.70	0.48-5.99	0.41
Adjusted for demographic, cardiovascular risk factors^c^	1.34	0.36-5.04	0.67
Adjusted for demographic, cardiovascular risk factors and eGFR^d^	0.84	0.12-6.17	0.87
Cardiovascular mortality			
Unadjusted^a^	15.90	3.52-71.87	<0.01
Adjusted for demographic factors^b^	10.0	2.13-47.19	<0.01
Adjusted for demographic, cardiovascular risk factors^c^	9.14	1.92-43.60	<0.01
Adjusted for demographic, cardiovascular risk factors and eGFR^d^	6.19	0.50-76.59	0.16

Abbreviations as in Table 1; HR = hazard ratio.

^a^All models additionally adjusted for hs-TnI.

^b^Adjusted for age and gender.

^c^Adjusted for model^b^ and hypertension, hyperlipidemia, smoking history and coronary heart disease.

^d^Adjusted for model^c^ and eGFR.

In order to evaluate the clinical application of hs-TnI level, the sensitivity, specificity, positive predictive value and negative predictive value of an elevated serum hs-TnI level was determined to predict future MACE. In patients with T2DM, an elevated serum hs-TnI level had a high specificity (84.4%) but limited sensitivity (62.7%) and positive predictive value (38.5%) for MACE. Conversely, a normal serum hs-TnI level had an excellent negative predictive value (92.2%) for future MACE in T2DM patients.

## Discussion

The present study demonstrates that up to 25% of T2DM patients without clinical evidence of active cardiovascular disease have ongoing subtle myocardial injury/necrosis as detected by an elevated serum level of hs-TnI. An elevated serum hs-TnI level in patients with T2DM, above the gender-specific cut-off value in the control population, was associated with a more than 2-fold increase in the adjusted risk of MACE. Importantly, a normal serum hs-TnI level had an excellent negative predictive value for future adverse cardiovascular outcome in patients with T2DM after up to 4 years of follow-up.

Elevated high sensitivity troponin level suggested myocardial injury that has been shown to be related with arterial stiffening in patients with T2DM [20]. In addition to detect subtle myocardial necrosis, its clinical value has also been shown to provide important prognostic information in various groups of patients. Several studies have demonstrated the predictive value for future cardiovascular events by hs-TnT in the general population [4,5,21], patients with stable and unstable angina [22], acute coronary syndrome [23] and heart failure [24]. Similarly, hs-TnI has been shown to provide prognostic information in the general population [6], patients with stable atherosclerotic disease [10,11] and acute coronary syndrome [25]. Although these studies have consistently demonstrated that high sensitivity troponin can predict future adverse cardiovascular events in different groups of patients, its prognostic value has not been well studied in patients with T2DM. A recent report from the Women Health Study demonstrated that among diabetic women without cardiovascular disease (n = 512), a detectable hs-TnT was associated with total cardiovascular disease (adjusted hazard ratio [HR] = 1.76) and cardiovascular death (adjusted HR = 3.13) [12]. Conversely, another study using elevated hs-TnT above 99^th^ percentile reference could not demonstrate a statistical association with adverse cardiovascular outcome [14]. The contradictory results from these studies may suggest the potentially limited prognostic value of hs-TnT in patients with T2DM. Because of the different biological characteristics [26], the clinical relevance and strength of detecting myocardial injury between hs-TnT and hs-TnI may differ. Indeed, it has been shown that the prognostic value of hs-TnI may be superior to hs-TnT in a cohort of patients with stable coronary artery disease [11]. The prognostic implication of hs-TnI may thus be more robust than hs-TnT in patients with T2DM.

A prior study demonstrated that a low level of circulatory TnI (9 ng/L to 30 ng/L) was predictive of MACE (death, MI or stroke) in patients with T2DM who underwent elective coronary angiography [27]. Nonetheless no study has evaluated the predictive value for MACE using a high sensitivity assay of TnI (level of detection = 1.2 ng/L). The present study included an expanded and clinically relevant population, consisting of both male and female T2DM patients, with and without underlying cardiovascular disease. Our results demonstrate that elevated hs-TnI independently predicted MACE (adjusted HR = 2.85) in patients with T2DM, and thus provides further evidence that elevated hs-TnI is closely associated with and predictive for future adverse events in patients with T2DM.

In this study, the high prevalence of MACE in T2DM patients with elevated hs-TnI was mainly driven by more frequent heart failure and cardiovascular death. In contrast, an elevated hs-TnI was not associated with future myocardial infarction. Indeed, prior studies have shown that among patients with stable chronic cardiovascular disease, an elevated troponin level better predicts heart failure than ischemic events [2,4,5,7,11,21]. In the Framingham Offspring Study, an elevated hs-TnI was independently predictive of death, heart failure and major cardiovascular events, but not coronary heart disease [7]. Similarly, Omland et al. showed that among patients with stable coronary artery disease, an elevated hs-TnI was strongly associated with cardiovascular death and heart failure but only weakly with non-fatal myocardial infarction [11]. Collectively, these findings support the hypothesis that a measurable circulating troponin level reflects chronic myocardial damage/myocardial stress, rather than acute ischemic insult or vascular stress. It can thereby identify an increased risk for pathological cardiac remodeling and subsequent heart failure.

In patients with T2DM, a number of mechanisms might explain the presence of ongoing subtle myocardial injury, including coronary microvascular dysfunction [28], depletion of endothelial progenitor cells [9], elevated oxidative stress [9,28], and advanced glycation end-products [29]. This is further evidenced by a study that showed that chronic hyperglycaemia, as measured by HbA1c, was independently associated with subclinical myocardial injury in subjects without clinically evident coronary artery disease, as assessed by elevated levels of hs-TnT [13]. In addition, this study also showed that the association of HbA1c with hs-TnT extends to subjects below the diagnostic threshold of 6.5%, and suggests that hyperglycaemia-related myocardial injury may begin before the onset of clinically evident diabetes. The circulating troponin detected by these high sensitive assays in patients with T2DM thus represents an intermediate phenotype of subtle myocardial injury, rather than an acute ischemic event.

In patients with T2DM, a number of different cardiovascular investigations, including treadmill testing [30], computed tomography angiography [31] and electrocardiogram-gated single photon emission computed tomography [32] have been shown to provide excellent negative predictive value for future cardiovascular events. Nevertheless their widespread clinical use for risk stratification is limited by the need for an experienced operator; prolonged study duration and prohibitive cost. One of the most intriguing findings of the present report was that a normal hs-TnI had a high negative predictive value for future adverse cardiovascular events in patients with T2DM after up to four years of follow-up. The present study thus suggests that a single serum measurement of hs-TnI provides a simple and inexpensive means to accurately risk stratify patients with T2DM, particularly identifying those at low risk for intermediate-term (>4 years) adverse cardiovascular events.

Interpretation of both hs-TnI and hs-TnT level can be done using a cut-off value either above the level of detection or above the 99^th^ percentile of a reference population [2]. Even within the same study population, the proportion of subjects with detectable hs-TnI is greater than hs-TnT [15]. The present study has shown that >99% of patients with T2DM had a detectable hs-TnI, compared with only 45.5% of diabetic women in the Women’s Health Study using hs-TnT [12]. Further, up to 25% of patients with T2DM had an elevated hs-TnI defined according to the 99^th^ percentile of an age and gender matched reference population. This finding demonstrates that hs-TnI is a sensitive assay to detect subtle myocardial injury, and up to one quarter of stable patients with T2DM had significant myocardial damage. In addition to the prognostic implication, this assay hence can be used as a surrogate for subtle myocardial injury to further study the mechanism of myocardial damage in patients with T2DM.

### Limitations

The present study included only a small study population of patients with T2DM and future study with a larger number of subjects is required to confirm the results. None of the patients received further coronary work-up based on the hs-TnI level alone. Future studies should evaluate the predictive value of significant coronary artery disease in T2DM patients with elevated hs-TnI. The study population is Asian, thus it may not be appropriate to extrapolate the results to other ethnic groups. Finally, only a single measurement of hs-TnI was used for risk stratification. Whether serial measurements of hs-TnI would provide additional information requires further evaluation.

## Conclusion

This study demonstrated that elevated hs-TnI was associated with MACE, heart failure, myocardial infarction and cardiovascular mortality in patients with T2DM. In addition, elevated hs-TnI was independently associated with MACE and heart failure after multivariate adjustment. Importantly, a single measurement of hs-TnI provided a high negative predictive value for cardiovascular outcome in patients with T2DM.

## Competing interest

The authors declare that they have no competing interests.

## Authors’ contributions

YK: Design of study, data collection, drafted the manuscript, LK, ZC, CY, CY, ZZ, WA: Data collection, LC: Design of study, TH: Design of study, drafted the manuscript. All authors read and approved the final manuscript.
